# Supplementary material for: Asparagine and Glutamine Deprivation Alters Ionizing Radiation Response, Migration and Adhesion of a p53null Colorectal Cancer Cell Line
Source: Int J Mol Sci. 2023 Feb 3;24(3):2983. doi: 10.3390/ijms24032983 (PMC9917910; doi:10.3390/ijms24032983)
Supplement: Supplementary file 1 [file ijms-24-02983-s001.zip › ijms-2023862-supplementary.pdf]

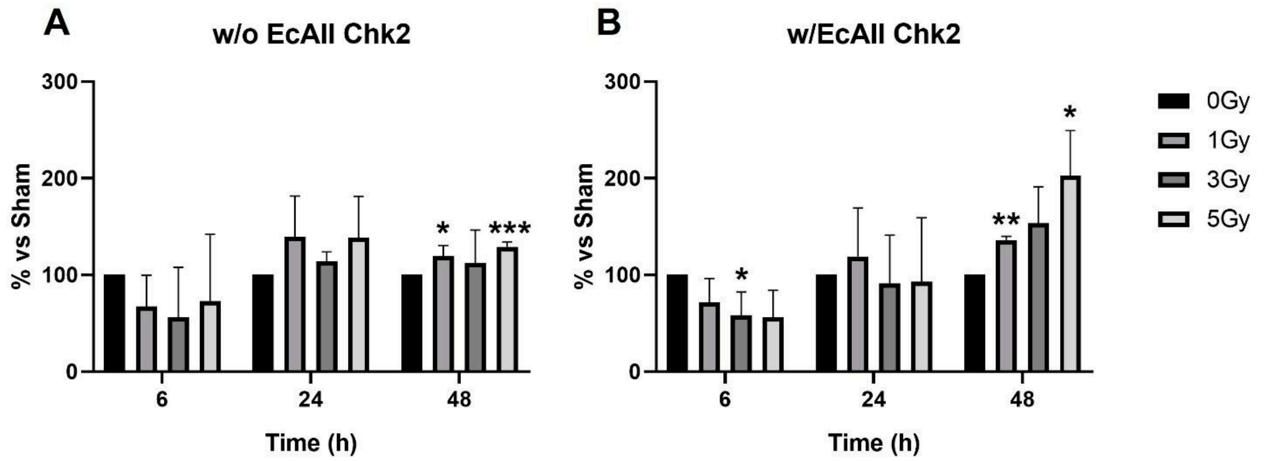

Figure S1. **G2/M transition phase protein analysis: Chk2 data normalized for the internal control.** The densitometric analysis was performed evaluating the p-Chk2/total Chk2 ratio, with respect to the unirradiated samples of each time point in absence of EcAII treatment (A) and with the administration of 1U/ml EcAII (B). Data reported are mean ± SD, obtained from at least 3 independent experiments. Statistical significance (Student's t-test) is calculated comparing the w/o and w/EcAII conditions for each dose and time point and reported as follows: *p < 0.05, ***p < 0.001.

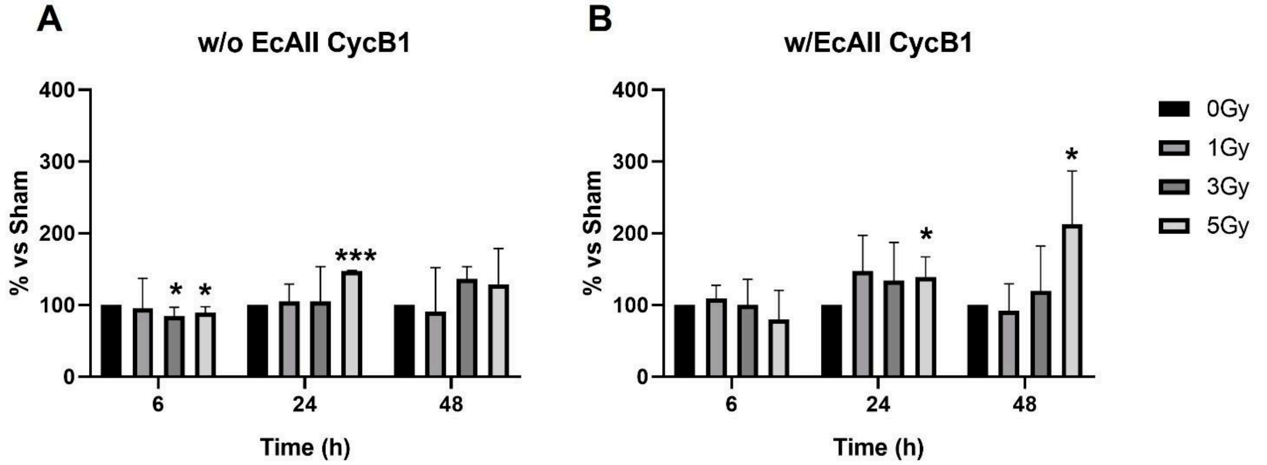

Figure S2. **G2/M transition phase protein analysis: CycB1 data normalized for the internal control.** The densitometric analysis was performed evaluating the CycB1 /GAPDH ratio, with respect to the unirradiated samples of each time point in absence of EcAII treatment (A) and with the administration of 1U/ml EcAII (B). Data reported are mean ± SD, obtained from at least 3 independent experiments. Statistical significance (Student's t-test) is calculated comparing the w/o and w/EcAII conditions for each dose and time point and reported as follows: *p < 0.05, **p < 0.01, ***p < 0.001.

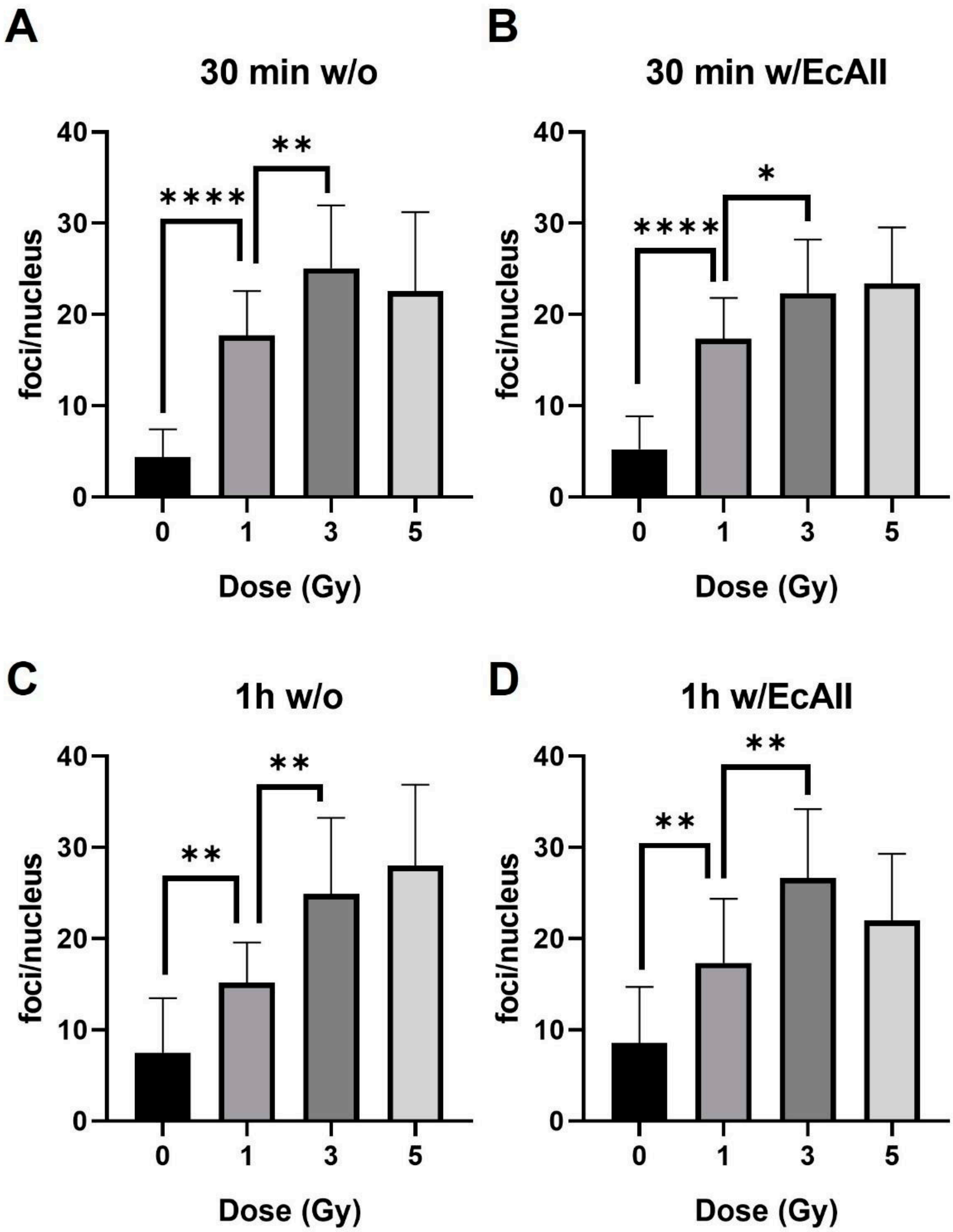

Figure S3. Count of γH2AX foci per nucleus. The foci scoring was analysed in absence of EcAII treatment at 30 min (A) and 1h (C) after X-ray exposure or with EcAII treatment at 30 min (B) and 1h (D) after X-ray exposure. Data reported are mean ± SD, obtained from at least 3 independent experiments. Statistical significance (Student's t-test) is calculated comparing the w/o and w/EcAII conditions for each dose and time point and reported as follows: *$p < 0.05$, ** $p < 0.01$, ****$p < 0.001$.

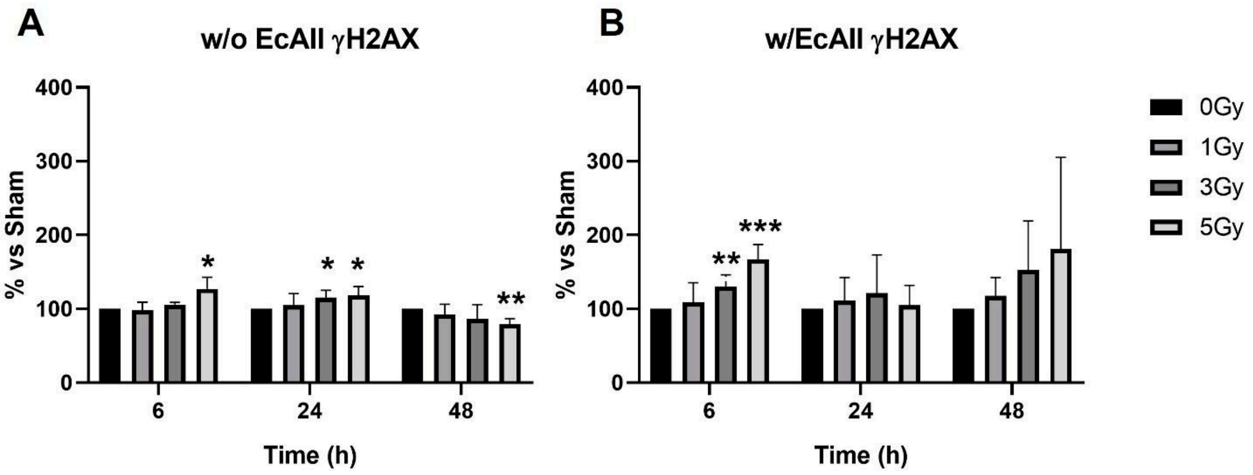

Figure S4. **DNA damage marker analysis: γH2AX data normalized for the internal control.** The densitometric analysis was performed evaluating the γH2AX/total H2AX ratio, with respect to the unirradiated samples of each time point in absence of EcAII treatment (A) and with the administration of 1U/ml EcAII (B). Data reported are mean ± SD, obtained from at least 3 independent experiments. Statistical significance (Student's t-test) is calculated comparing the w/o and w/EcAII conditions for each dose and time point and reported as follows: *p < 0.05, ** p < 0.01, ***p < 0.001.

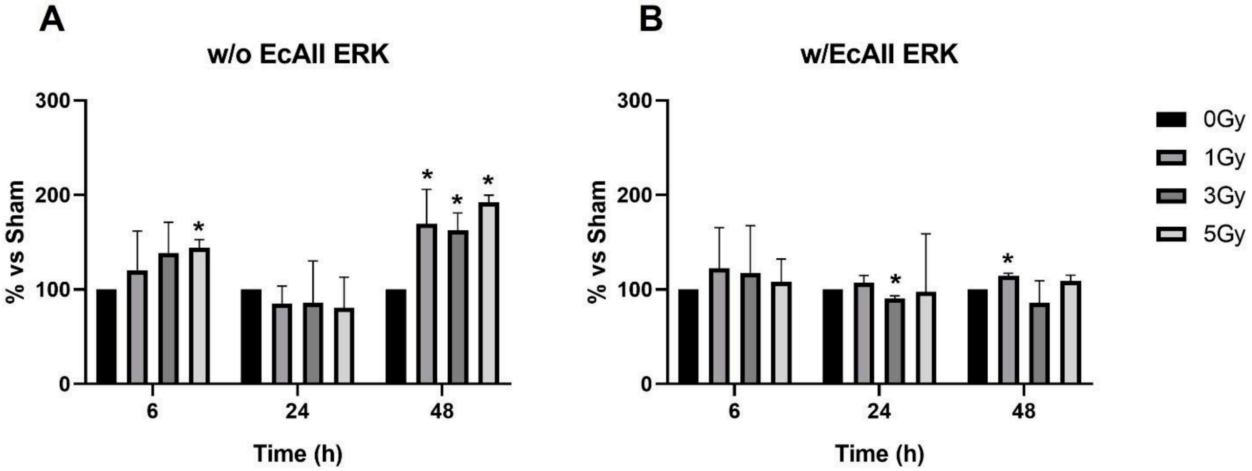

Figure S5. **Metabolic response evaluation: ERK data normalized for the internal control.** The densitometric analysis was performed evaluating the p-ERK/total ERK ratio, with respect to the unirradiated samples of each time point in absence of EcAII treatment (A) and with the administration of 1U/ml EcAII (B). Data reported are mean ± SD, obtained from at least 3 independent experiments. Statistical significance (Student's t-test) is calculated comparing the w/o and w/EcAII conditions for each dose and time point and reported as follows: *p < 0.05.

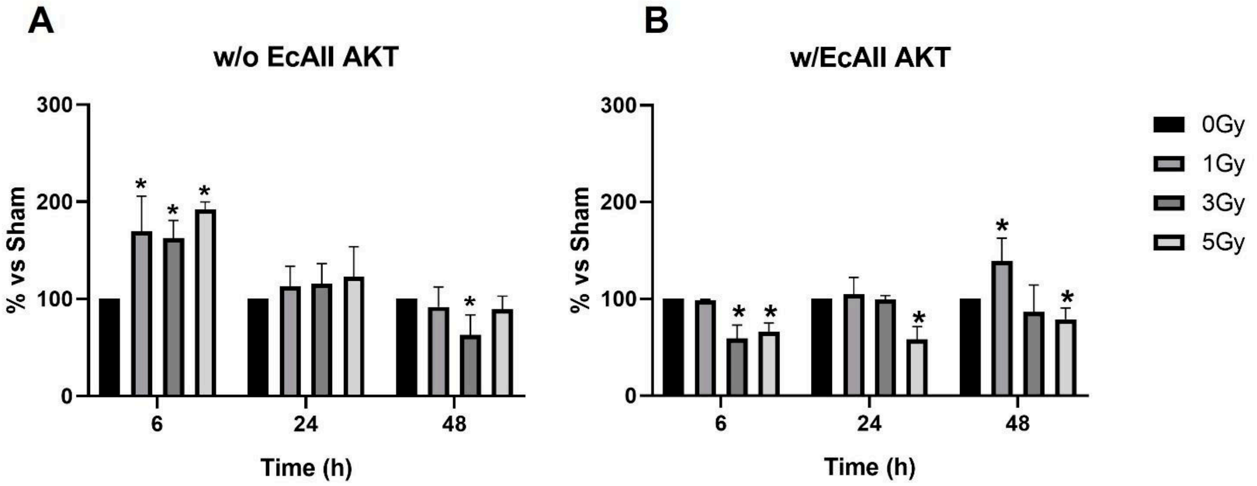

Figure S6. **Metabolic response evaluation: Akt data normalized for the internal control.** The densitometric analysis was performed evaluating the p-AKT/total AKT ratio, with respect to the unirradiated samples of each time point in absence of EcAII treatment (A) and with the administration of 1U/ml EcAII (B). Data reported are mean ± SD, obtained from at least 3 independent experiments. Statistical significance (Student's t-test) is calculated comparing the w/o and w/EcAII conditions for each dose and time point and reported as follows: *$p < 0.05$.
